# Age-Dependent Activation of Pannexin1 Function Contributes to the Development of Epileptogenesis in Autosomal Dominant Sleep-related Hypermotor Epilepsy Model Rats

**DOI:** 10.3390/ijms25031619

**Published:** 2024-01-28

**Authors:** Kouji Fukuyama, Eishi Motomura, Motohiro Okada

**Affiliations:** Department of Neuropsychiatry, Division of Neuroscience, Graduate School of Medicine, Mie University, Tsu 514-8507, Japan; k-fukuyama@clin.medic.mie-u.ac.jp (K.F.); motomura@clin.medic.mie-u.ac.jp (E.M.)

**Keywords:** ADSHE, astrocytes, epileptogenesis, hemichannel, ictogenesis, pannexin1

## Abstract

To explore the processes of epileptogenesis/ictogenesis, this study determined the age-dependent development of the functional abnormalities in astroglial transmission associated with pannexin1-hemichannel using a genetic rat model of autosomal dominant sleep-related hypermotor epilepsy (ADSHE) named ‘S286L-TG’. Pannexin1 expression in the plasma membrane of primary cultured cortical astrocytes and the orbitofrontal cortex (OFC), which is an ADSHE focus region, were determined using capillary immunoblotting. Astroglial D-serine releases induced by artificial high-frequency oscillation (HFO)-evoked stimulation, the removal of extracellular Ca^2+^, and the P2X7 receptor agonist (BzATP) were determined using ultra-high performance liquid chromatography (UHPLC). The expressions of pannexin1 in the plasma membrane fraction of the OFC in S286L-TG at four weeks old were almost equivalent when compared to the wild type. The pannexin1 expression in the OFC of the wild type non-statistically decreased age-dependently, whereas that in S286L-TG significantly increased age-dependently, resulting in relatively increasing pannexin1 expression from the 7- (at the onset of interictal discharge) and 10-week-old (after the ADSHE seizure onset) S286L-TG compared to the wild type. However, no functional abnormalities of astroglial pannexin1 expression or D-serine release through the pannexin1-hemichannels from the cultured astrocytes of S286L-TG could be detected. Acutely HFO-evoked stimulation, such as physiological ripple burst (200 Hz) and epileptogenic fast ripple burst (500 Hz), frequency-dependently increased both pannexin1 expression in the astroglial plasma membrane and astroglial D-serine release. Neither the selective inhibitors of pannexin1-hemichannel (10PANX) nor connexin43-hemichannel (Gap19) affected astroglial D-serine release during the resting stage, whereas HFO-evoked D-serine release was suppressed by both inhibitors. The inhibitory effect of 10PANX on the ripple burst-evoked D-serine release was more predominant than that of Gap19, whereas fast ripple burst-evoked D-serine release was predominantly suppressed by Gap19 rather than 10PANX. Astroglial D-serine release induced by acute exposure to BzATP was suppressed by 10PANX but not by Gap19. These results suggest that physiological ripple burst during the sleep spindle plays important roles in the organization of some components of cognition in healthy individuals, but conversely, it contributes to the initial development of epileptogenesis/ictogenesis in individuals who have ADSHE vulnerability via activation of the astroglial excitatory transmission associated with pannexin1-hemichannels.

## 1. Introduction

Recently, functional abnormalities in tripartite synaptic transmission or astroglial excitatory transmission have been considered to play important roles in the development of epileptogenesis and/or ictogenesis [1,2,3,4,5,6,7,8]. Functional abnormalities of astroglial hemichannels containing connexin43 [9,10,11,12,13] have been demonstrated in a validated genetic rat model of autosomal dominant sleep-related hypermotor epilepsy (ADSHE), named “S286L-TG”; it bears the missense S286L-mutant rat *Chrna4*, which corresponds to the S284L-mutant human *CHRNA4* of patients with ADSHE [8,14]. It is critical evidence that loss of function of the S284L-mutant α4 subunit of nicotinic acetylcholine receptors (α4-nAChR) plays a primary role in the initial stages of the development of ADSHE in epileptogenesis [8,14]; however, α4-nAChR selectively expresses in neurons but not in astrocytes [14]. These discrepancies between the expression of mutant α4-nAChR in neurons and the observed major functional abnormalities in astrocytes suggest that the epileptogenesis/ictogenesis of ADSHE is probably composed of complexes among the various age-dependent functional abnormalities [8,11,12,14].

Some of the mechanisms of epileptogenesis/ictogenesis in S286L-TG that have been revealed so far are as follows. Initially, the loss of function of mutant nAChR leads to GABAergic disinhibition in the thalamus, resulting in activation of glutamatergic neurons from the thalamus to various projecting regions [9,10]. In particular, under the GABAergic disinhibition, thalamic glutamatergic neurons also propagate excitabilities with physiological sleep spindle bursts to the cortex and basal ganglia [9,10]. Accumulating propagated excitabilities accelerate tripartite synaptic transmission in the frontal cortex and basal ganglia through the activation of astroglial connexin43-hemichannels [12,13], resulting in the enhancement of the intracellular signaling of both extracellular signal-regulated kinase (Erk) and protein kinase B (Akt) [8,11,12]. Combinations of enhanced electrophysiological excitability and activated signaling of Erk/Akt accelerate the trafficking of connexin43 to the plasma membrane [8,9,10,12,13]. Finally, the synergic interactions between hyperactivated astroglial hemichannels, GABAergic disinhibition, enhanced signaling of Erk/Akt, and sleep-related high-frequency oscillations (HFOs) during sleep spindle bursts (ripple bursts) lead to the generation of epileptogenic fast ripple bursts [11,12]. These findings suggest that functional abnormalities in neurotransmission and tripartite synaptic transmission play fundamental roles in the development of epileptogenesis/ictogenesis in ADSHE patients with S284L-mutation or S286L-TG [8]. Considering the recently reported findings regarding the functional abnormalities in tripartite synaptic transmission for development processes in the epileptogenesis/ictogenesis of S286L-TG and exploring the possibility that functional abnormalities of pannexin1 are also involved in the developmental processes in the epileptogenesis/ictogenesis of S286L-TG can provide the basis for understanding the pathomechanisms of not only ADSHE but also epilepsy.

Pannexin1 and connexin43 do not share homologies in their peptide sequences, whereas connexin43 and pannexin1 are assembled in hexamers to form connexon and pannexon in the plasma membrane, respectively [15,16,17]. The topologies and structures between connexon and pannexon in proteins comprise a similar group of transmembrane pores, which are permeable to ions, second messengers, and several signaling mediators up to 1.5 kDa [4,5,16,17,18]. Therefore, both connexon and pannexon serve as routes of ionic and large molecular interchange between the cytoplasm and the extracellular compartment [16,17]; however, connexon also forms the pore for the gap junction between the cytoplasm of two adjacent cells, but pannexon is considered to be unable to constitute gap junctions [19,20,21]. Furthermore, during the resting stage, connexin43-hemichannels exhibit low opening probability during the resting stage, whereas pannexin1-hemichannel can open due to its lower negative threshold potentials [22,23]. Both connexin43-hemichannels and pannexin1-hemichannels are activated by depolarization, but pannexin1-hemichannels can reach to maximum currents with faster kinetics [22,24]. Furthermore, the gating properties of connexin43-hemichannels are regulated by extracellular Ca^2+^ dependency (the physiological range of the extracellular Ca^2+^ level inhibits connexin43-hemichannel opening probability); however, the pannexin1-hemichannel opening is independent of the extracellular Ca^2+^ level, but it is activated by increasing the intracellular Ca^2+^ level [23,25,26,27,28]. Additionally, sustained/repetitive exposure to adenosine triphosphate (ATP) leads to the formation of functional complexes between the P2X7 receptor (P2X7R) and the pannexin1-hemichannels, resulting in the transformation of the features of P2X7R from cation channels to non-selective channels permeable to large molecules [29,30,31].

These functional features of pannexin1-hemichannels can provide a candidate hypothesis regarding the pathomechanisms of ADSHE in S286L-TG. The activation of pannexin1-hemichannels induced by HFOs may make an earlier and more sensitive contribution to the development of epileptogenesis in S286L-TG compared to connexin 43 hemichannels, since pannexin1-hemichannels are more sensitive to depolarization and are independent of extracellular Ca^2+^ compared to connexin43-hemichannels. In other words, the functional abnormalities of pannexin1-hemichannels probably play fundamental roles in the development of the epileptogenesis/ictogenesis of ADSHE in S286L-TG. Based on our hypothesis, therefore, we determined the age-dependent fluctuation of pannexin1 expression in the plasma membrane and development of functional abnormalities associated with astroglial pannexin1-hemichannels in S286L-TG.

## 2. Results

### 2.1. Age-Dependent Expression of Pannexin1 in the Plasma Membrane in Orbitofrontal Cortex (OFC) of Wild Type and S286L-TG

The expressions of connexin43 in the wild type and in S286L-TG at 4 weeks of age (before the onset of interictal discharge [8,14]) are almost equal, but at 7 weeks of age (after the onset of interictal discharge) or 10 weeks of age (after the onset of ADSHE seizure), those in S286L-TG increased compared to the wild type [11,12]. Therefore, the age-dependent expressions of pannexin1 in the OFC (an ADSHE focus region) of the wild type and S286L-TG were determined using capillary immunoblotting.

**Figure 1:**
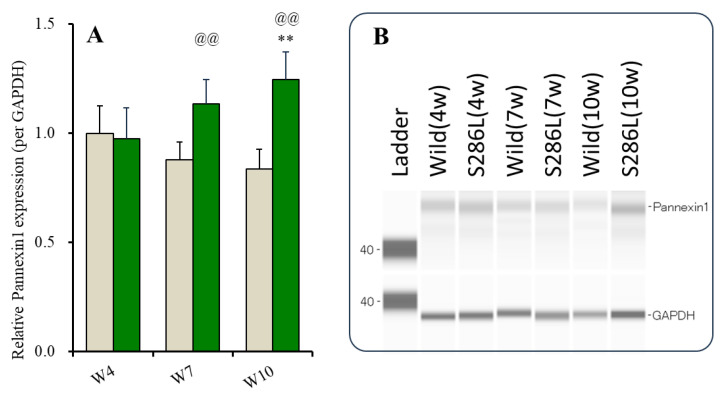
Age-dependent fluctuations of pannexin1 expression in the plasma membrane in OFC of wild type and S286L-TG. Panel (**A**) indicates expressions of pannexin1 in the plasma mem-brane fraction of OFC of wild type (brown columns) and S286L-TG (green columns) at 4, 7, and 10 weeks of age, respectively. Ordinates indicate the mean ± SD (n = 6) of relative expression of pannexin1 per GAPDH, and abscissas indicate ages (weeks). ** *p* < 0.01, relative to pannexin1 expression at 4 weeks of age, @@ *p* < 0.01, relative to the wild type of the same age using two-way ANOVA with Scheffe’s post hoc test. F value was [F_age_(2,30) = 0.7 (*p* > 0.1), F_genotype_(1,30) = 30.7 (*p* < 0.01), F_age*genotype_(2,30) = 11.0 (*p* < 0.01)]. Panel (**B**) indicates the pseudo-gel images of pannexin1 and GAPDH using capillary immunoblotting.

The expression of pannexin1 in the OFC of the wild type non-significantly and age-dependently decreased, whereas that of S286L-TG increased age-dependently from 4 to 10 weeks of age (Figure 1). In particular, the pannexin1 expressions at 4 weeks of age in the wild type and in S286L-TG were almost equal, but at 7 and 10 weeks of age, those of S286L-TG increased compared to the wild type (Figure 1).

In S286L-TG, the onset of interictal discharges was from 6 to 8 weeks of age, and ADSHE seizures were observed after 8 weeks of age [8,14]. Considering the phenotypic features of S286L-TG, these results suggested that pannexin1 was a candidate pathological molecule associated with epileptogenesis and/or ictogenesis in S286L-TG, since increasing pannexin1 expression in the OFC of S286L-TG was synchronized with the epileptic events, the onset of interictal discharge (from 6 to 8 weeks of age), and ADSHE seizure (after 8 weeks of age).

### 2.2. Expression of Pannexin1 on the Astroglial Plasma Membrane in Wild Type and S286L-TG

**Figure 2:**
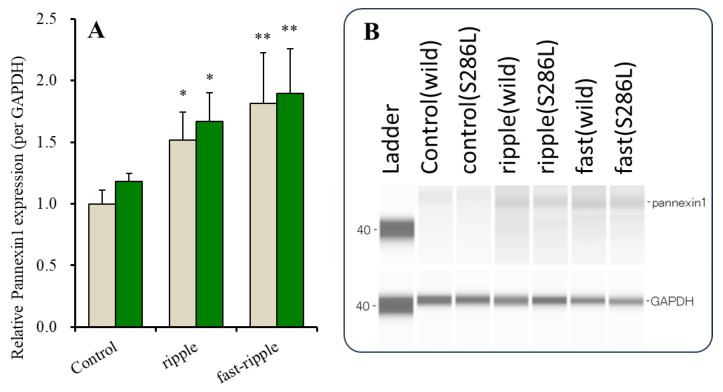
Astroglial expression in the plasma membrane evoked by ripple burst and fast ripple burst stimulations in wild type and S286L-TG. Panel (**A**) indicates expression of pannexin1 in the astroglial plasma membrane fraction. Ordinates indicate the mean ± SD (n = 6) of relative expression of pannexin1 per GAPDH. Gray and green bars indicate pannexin1 expression in astrocytes of wild type and S286L-TG after chronic fast ripple-evoked stimulation, respectively. * *p* < 0.05, ** *p* < 0.01 using two-way ANOVA with Scheffe’s post hoc test. F value was [F_genotype_(1,20) = 16.6 (*p* < 0.01), F_age_(1,20) = 2.0 (*p* > 0.1), F_genotype*age_(1,20) = 5.4 (*p* < 0.05)]. Panel (**B**) indicates the pseudo-gel images of P2X7R and GAPDH, using capillary immunoblotting.

The expressions of connexin43 in the astroglial plasma membrane in the wild type and S286L-TG have been reported to be almost equal [8,12]. Therefore, the expressions of pannexin1 in the plasma membrane fraction of the primary cultured cortical astrocytes of the wild type and S286L-TG were also determined using capillary immunoblotting. The basal expressions of pannexin1 in the plasma membrane fraction in the cultured astrocytes from the wild type and S286L-TG were also almost equal (Figure 2).

Chronic exposure (for 7 days) to HFO-evoked stimulations, such as physiological ripple burst [32,33,34] (10 stimuli at 200 Hz and 10 bursts (50% duty cycle) at burst intervals of 100 msec/sec) [11] and epileptogenic fast ripple burst [35,36,37] (10 stimuli at 500 Hz and 10 bursts (50% duty cycle) at burst intervals of 40 msec/sec) [11] frequency-dependently increased the expression of connexin43 in the astroglial plasma membrane via activation of its trafficking to the plasma membrane induced by the signaling of Erk and Akt [11].

Therefore, the effects of chronic (for 7 days) ripple burst- and fast ripple burst-evoked stimulations in the expression of pannexin1 in the astroglial plasma membrane of the wild type and S286L-TG were also determined using capillary immunoblotting. The expression of pannexin1 in the astroglial plasma membrane of both the wild type and S286L-TG also increased frequency-dependently, since the increasing pannexin1 expression evoked by fast ripple burst was larger than those evoked by ripple burst-evoked stimulation (Figure 2).

These results indicate that astroglial pannexin1 expression is possibly increased by chronic exposure to hyperexcitability. Therefore, astroglial pannexin1 is a candidate target molecule for the ADSHE epileptogenesis/ictogenesis of S286L-TG, since the astroglial pan-nexin1 expression in the plasma membrane was increased by HFO frequency-dependently and was similar to that of connexin43 [11].

### 2.3. Astroglial Transmission Associated with HFOs

#### 2.3.1. Effects of Acute HFO-Evoked Stimulations on D-Serine Releases from Cultured Astrocytes between Wild Type and S286L-TG

**Figure 3:**
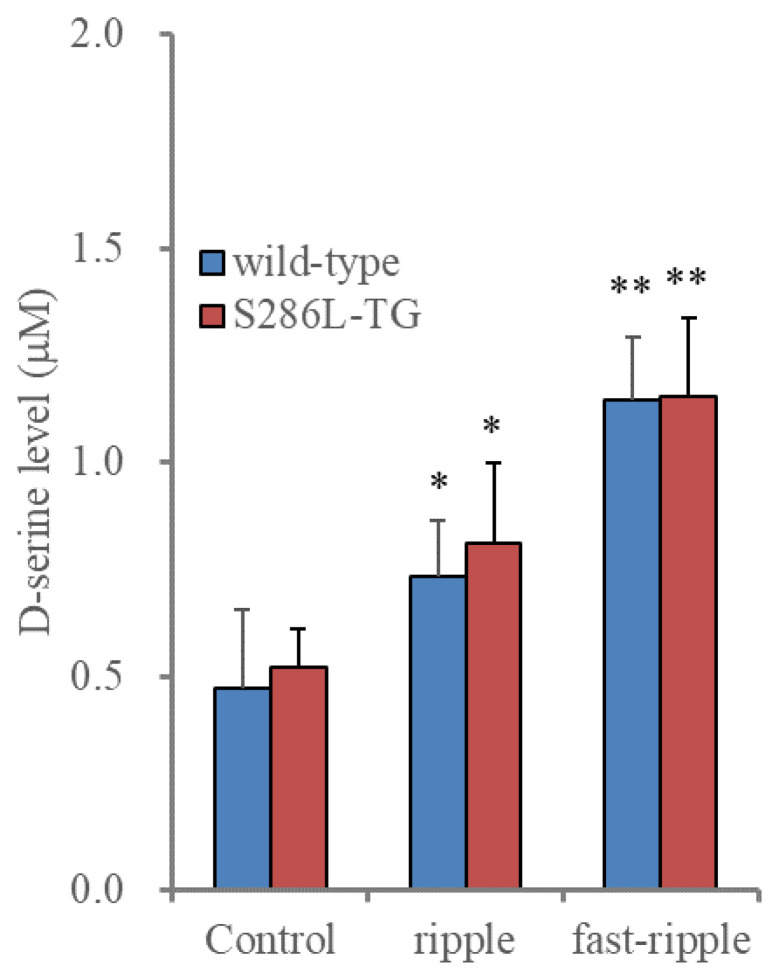
Acutely artificial high-frequency oscillation burst (HFO)-evoked releases of D-serine from cultured astrocytes of wild type and S286L-TG. Histogram represents the effects of HFO-evoked stimulation (ripple burst and fast ripple burst) on D-serine releases from cultured astrocytes of wild type (blue) and S286L-TG (red). Ordinates indicate the mean ± SD (n = 6) of extracellular D-serine level (μM) during the ripple burst- and fast ripple burst-evoked stimulation. * *p* < 0.05, ** *p* < 0.01 relative to control (basal release: non-stimulation) using two-way analysis of variance (ANOVA) with Scheffe’s post hoc test. F value was [F_HFO_(2,30) = 51.0 (*p* < 0.01), F_genotype_(1,30) = 0.7 (*p* > 0.1), F_HFO*genotype_(2,30) = 0.2 (*p* > 0.1)].

In the above study, the abnormalities of the pannexin1 expression in the astrocytes of S286L-TG could not be detected when compared to the wild type. Therefore, consequently, to explore the functional abnormalities in the astroglial transmission in S286L-TG, the release of D-serine, a major gliotransmitter, from cultured astrocytes of the wild type and S286L-TG was determined. The basal astroglial release of D-serine was almost equal in the wild type and S286L-TG (Figure 3).

It has been already revealed that the astroglial release of L-glutamate was frequency-dependently increased by artificial HFO-evoked stimulation [11]. Based on the previous findings, the D-serine releases induced by HFO-evoked stimulation from cultured astrocytes in the wild type and S286L-TG were also determined. As with L-glutamate, any differences in the D-serine release from cultured astrocytes between the wild type and S286L-TG induced by acute HFO-evoked stimulations, including ripple burst- and fast ripple-evoked stimulations [11], could not be detected.

These results suggest that the astrocytes in S286L-TG possibly have no functional abnormalities in the astroglial transmissions directly induced by genetic abnormalities in *Chrna4*, since the differences in both the pannexin1 expression and the HFO-evoked D-serine release could not be detected. Thus, to clarify, the mechanisms underlying the increasing pannexin1 expression and enhancing the tripartite synaptic transmission in the OFC were investigated using cultured astrocytes of the wild type.

#### 2.3.2. Effects of Extracellular Ca^2+^ Removal on Astroglial HFO-Evoked D-Serine Release

It has been demonstrated that pannexin1 and connexin43 form unopposed functional hemichannels in astrocytes [38,39,40,41,42]. Astroglial gliotransmitter release through connexin43-hemichannels was enhanced by decreasing the extracellular Ca^2+^ level, whereas that through pannexin1-hemichannels was probably independent of the extracellular Ca^2+^ level [23,27,28]. Exocytotic transmitter release is dependent on the extracellular Ca^2+^ level [43]. Therefore, the functional features of gliotransmitter release through the connexin43-hemichannels and pannexin1-hemichannels are quite different from those of exocytotic transmitter release. Based on these findings, to explore the mechanisms of HFO-evoked astroglial D-serine release, the effects of the removal of the extracellular Ca^2+^ level on HFO-evoked astroglial D-serine release were determined.

**Figure 4:**
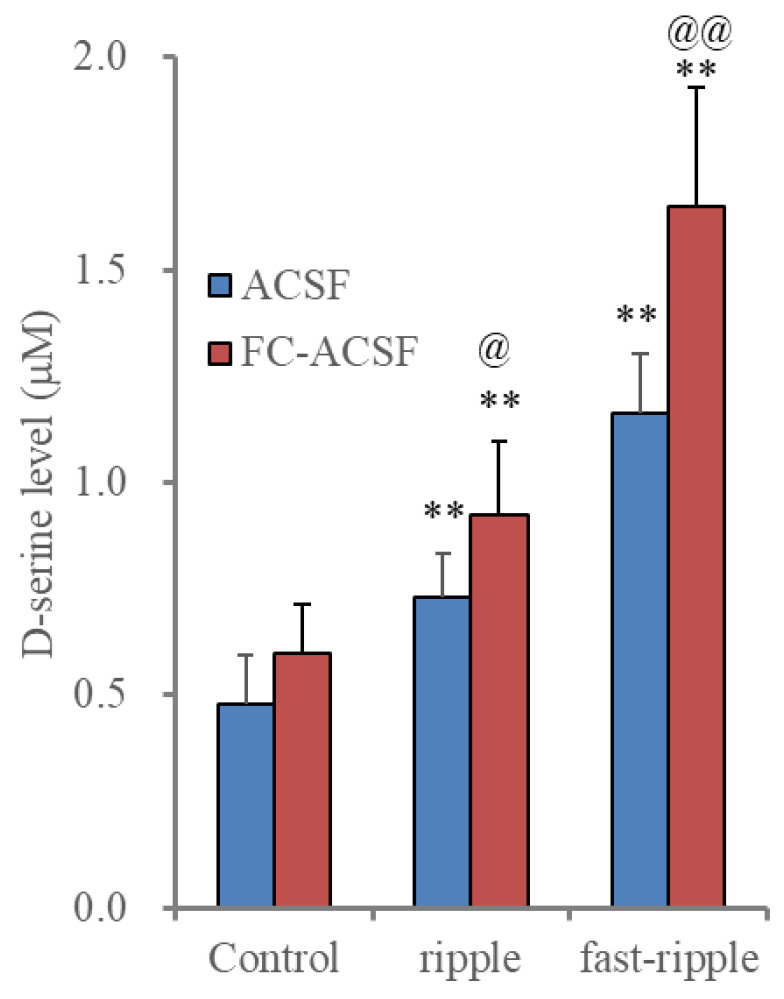
Effects of removal extracellular Ca^2+^ on astroglial HFO-evoked D-serine release. Histogram represents the HFO-evoked astroglial D-serine release from cultured astrocytes of wild type incubated in normal artificial cerebrospinal fluid (ACSF: blue) and Ca^2+^-free artificial cerebrospinal fluid (FC-ACSF: red). Ordinates indicate the mean ± SD (n = 6) of extracellular D-serine level (μM) during the ripple burst- and fast ripple burst-evoked stimulation. ** *p* < 0.01 relative to control (basal release: non-stimulation), @ *p* < 0.05, @@ *p* < 0.01 relative to ACSF using multivariate analysis of variance (MANOVA) with Scheffe’s post hoc test. F value was [F_HFO_(2,20) = 314.8 (*p* < 0.01), F_ACSF_(1,10) = 9.4 (*p* < 0.05), F_HFO*ACSF_(2,20) = 15.0 (*p* < 0.01)].

Incubation in a Ca^2+^-free artificial cerebrospinal fluid (FC-ACSF) enhanced HFO-evoked astroglial D-serine release without affecting basal release, compared to incubation in normal artificial cerebrospinal fluid (ACSF) (Figure 4). Additionally, the stimulatory effects of the removal of the extracellular Ca^2+^ level were enhanced by HFO-evoked stimulations frequency-dependently (Figure 4).

Although the energization amounts of the ripple-evoked and fast ripple-evoked stimulations were set to be equivalent, the fast ripple-evoked stimulation predominantly increased astroglial D-serine release compared to ripple-evoked stimulation. Therefore, astroglial D-serine release was dependent on the decreasing extracellular Ca^2+^ level and the frequency of the depolarizations.

#### 2.3.3. Effects of Selective Inhibitors of Connexin43 and Pannexin1 on HFO-Evoked Astroglial D-Serine Release

To identify the fundamental molecules underlying increased astroglial D-serine release induced by HFO-evoked stimulation and the removal of extracellular Ca^2+^, the effects of the selective inhibitors of connexin43, the N-terminal transactivator of transcription Gap19 (Gap19) [44], and pannexin1, 10PANX [31,45], on astroglial D-serine release were determined.

**Figure 5:**
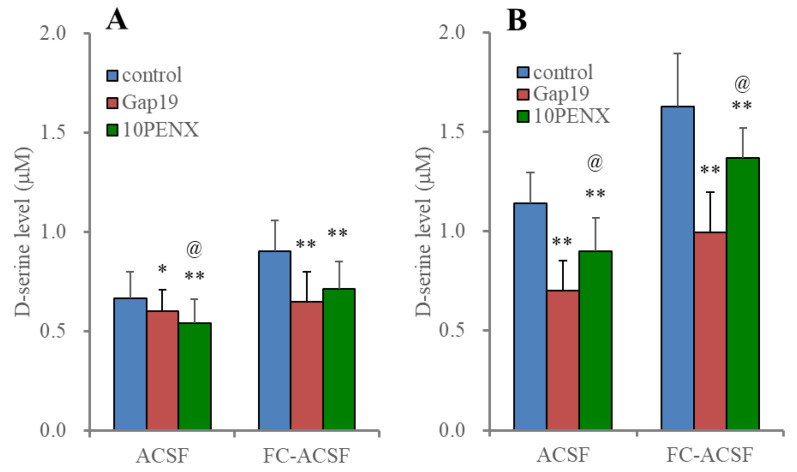
Effects of Gap19 and 10PANX on HFO-evoked astroglial D-serine release. Panel A indicates the effects of inhibitors of connexin43 (Gap19) and pannexin1 (10PANX) on ripple burst-evoked astroglial D-serine releases in ACSF and FS-ACSF. Panel B indicates the effects of Gap19 and 10PANX on fast ripple burst-evoked astroglial D-serine releases in ACSF and FS-ACSF. Ordinates indicate the mean ± SD (n = 6) of extracellular D-serine level (μM) during the ripple burst- and fast ripple burst-evoked stimulation. * *p* < 0.05, ** *p* < 0.01 relative to control, @ *p* < 0.05 relative to Gap19, using MANOVA with Scheffe’s post hoc test. F values in effects of ripple-evoked stimulation in ACSF and FC-ACSF on D-serine release from cultured astrocytes (in panel (**A**)) were [F(2,10) = 29.4 (*p* < 0.01)] and [F(2,10) = 26.4 (*p* < 0.01)] using MANOVA, respectively. F values in effects of fast ripple-evoked stimulation in ACSF and FC-ACSF on D-serine release from cultured astrocytes (in panel (**B**)) were [F(1.0,5.0) = 135.6 (*p* < 0.01)] and [F(2,10) = 77.0 (*p* < 0.01)] using MANOVA, respectively.

According to our speculation, both 20 μM Gap19 and 100 μM 10PANX suppressed HFO-evoked D-serine release (Figure 5). The inhibitory effects of 10PANX on ripple burst-evoked D-serine release incubated in ACSF were predominant compared to Gap19; however, fast ripple-evoked stimulation in either ACSF or FC-ACSF was suppressed by Gap19 predominantly rather than by 10PANX (Figure 5). The inhibitory effects of Gap19 and 10PANX on ripple-evoked D-serine release in FC-ACSF were almost equal (Figure 5).

These results suggest that the gating properties of the connexin43-hemichannels are more sensitive to the decreasing extracellular Ca^2+^ level compared to the pannexin1-hemichannels [23,25,26,27,28], whereas the sensitivity to membrane depolarization of the pannexin1-hemichannels is shown to be dominant compared to the connexin43-hemichannels [22,24,44].

#### 2.3.4. Effects of Hemichannel Inhibitors on HFO-Evoked Astroglial D-Serine Release

It has been established that probenecid and carbenoxolone are non-peptide hemichannel inhibitors [2,45,46,47]. Probenecid is a relatively selective pannexin1-hemichannel inhibitor [46,48], whereas carbenoxolone is a non-selective inhibitor for the pannexin1-hemichannel, connexin43-hemichannel, gap junctions, and other channels [12,49,50,51,52,53,54]. To identify the fundamental molecules underlying increased astroglial D-serine release induced by HFO-evoked stimulation and the removal of extracellular Ca^2+^, the concentration-dependent effects of probenecid and carbenoxolone on astroglial D-serine release were also determined.

**Figure 6:**
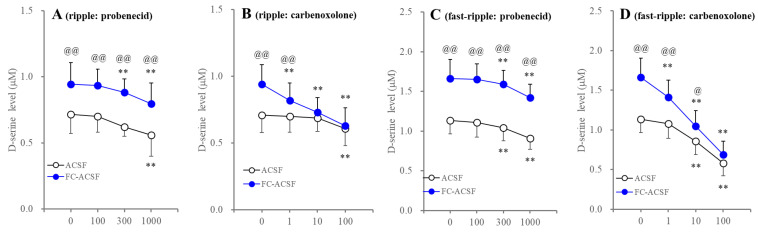
Concentration-dependent effects of probenecid and carbenoxolone on HFO-evoked astroglial D-serine release. Panels (**A**,**C**) indicate the concentration-dependent effects of probenecid (from 100 to 1000 μM) on ripple-evoked and fast ripple-evoked astroglial D-serine releases in ACSF (opened circles) and FC-ACSF (blue circles), respectively. Panels (**B**,**D**) indicate the concentration-dependent effects of carbenoxolone (from 1 to 100 μM) on ripple-evoked and fast ripple-evoked astroglial D-serine releases in ACSF (opened circles) and FC-ACSF (blue circles), respectively. Ordinates indicate the mean ± SD (n = 6) of extracellular D-serine level (μM) during the ripple burst- and fast ripple burst-evoked stimulation. Abscissas indicate concentration of probenecid and carbenoxolone (μM). ** *p* < 0.01 relative to control (0 μM), @ *p* < 0.05, @@ *p* < 0.01 relative to ACSF, using MANOVA with Scheffe’s post hoc test. F values in effects of probenecid on D-serine release in panels (**A**,**C**) were [F_probenecid_(3,30) = 21.1 (*p* < 0.01), F_ACSF_(1,10) = 10.9 (*p* < 0.01), F_probenecid*ACSF_(3,30) = 0.3 (*p* > 0.1)] and [F_probenecid_(3,30) = 49.5 (*p* < 0.01), F_ACSF_(1,10) = 27.2 (*p* < 0.01), F_probenecid*ACSF_(3,30) = 0.3 (*p* > 0.1)], respectively. F values in effects of carbenoxolone on D-serine release in panels (**B**,**D**) were [F_carbenoxolone_(3,30) = 71.7 (*p* < 0.01), F_ACSF_(1,10) = 2.1 (*p* < 0.01), F_carbenoxolone*ACSF_(3,30) = 21.6 (*p* < 0.01)] and [F_carbenoxolone_(3,30) = 338.9 (*p* < 0.01), F_ACSF_(1,10) = 7.5 (*p* < 0.05), F_carbenoxolone*ACSF_(3,30) = 25.5 (*p* < 0.01)], respectively.

Both probenecid and carbenoxolone concentration-dependently suppressed HFO-evoked astroglial D-serine release (Figure 6). In particular, 100 μM carbenoxolone abolished the enhanced ripple-evoked and fast ripple-evoked D-serine releases induced by the removal of extracellular Ca^2+^; however, probenecid did not affect the stimulatory effects of removal of extracellular Ca^2+^ on HFO-evoked D-serine releases (Figure 6). These results suggest that decreasing extracellular Ca^2+^ activates the connexin43-hemichannel but not the pannexin1-hemichannels.

### 2.4. Effects of Inhibitors of Connexin43 and Pannexin1 on Astroglial D-Serine Release Induced by P2X7R Activation

It is well known that the activation of P2X7R enhances astroglial gliotransmitter release via the enhanced opening of the P2X7R and pannexin1-hemichannels [31,55]. P2X7R was co-immunoprecipitated with pannexin1 as part of the P2X7R/pannexin1 complex [30,56]. Additionally, the P2X7R agonist-evoked ATP release was mediated through the pannexin1-hemichannels in astrocyte [57,58]. Therefore, the effects of the inhibitors of connexin43, pannexin1, and P2X7R on astroglial D-serine release induced by acute exposure to the P2X7R agonist, 2′(3′)-O-(4-benzoylbenzoyl)adenosine-5′-triphosphate tri(triethylammonium) salt (BzATP), were determined.

**Figure 7:**
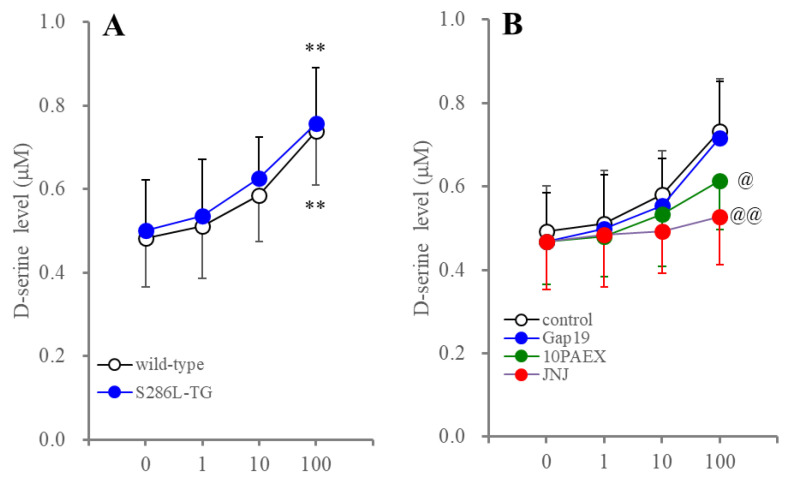
Effects of BzATP-evoked stimulation on astroglial D-serine release. Panel (**A**) indicates the concentration-dependent effects of BzATP (P2X7R agonist from 1 to 100 μM) on D-serine release from cultured astrocytes between wild type and S286L-TG. Panel (**B**) indicates the effects of selective inhibitors of connexin43-hemichannel (Gap19), pannexin1-hemichannel (10PANX), and P2X7R (JNJ: JNJ47965567) on BzATP-evoked astroglial D-serine release. Ordinates indicate the mean ± SD (n = 6) of extracellular D-serine level (μM) during the BzATP-evoked stimulation. In panel (**A**): ** *p* < 0.01 relative to control (BzATP free) using two-way ANOVA with Scheffe’s post hoc test. F value in panel (**A**) was [F_BzATP_(3,40) = 10.5 (*p* < 0.01), F_genotype_(1,40) = 0.5 (*p* > 0.1), F_BzATP*genotype_(3,40) = 0.1 (*p* > 0.1)]. In panel (**B**): @ *p* < 0.05, @@ *p* < 0.01 relative to control (without inhibitors) using MANOVA with Scheffe’s post hoc test. F value was [F_BzATP_(3,60) = 105.2 (*p* < 0.01), F_inhibitor_(3,20) = 0.7 (*p* > 0.1), F_BzATP*inhibitor_(9,60) = 7.5 (*p* > 0.1)].

BzATP concentration-dependently increased D-serine release from the cultured astrocytes of both the wild type and S286L-TG (Figure 7). BzATP-evoked astroglial D-serine release was completely inhibited by the selective P2X7R antagonist, 3 μM 2-(phenylthio)-*N*-[[tetrahydro-4-(4-phenyl-1-piperazinyl)-2H-pyran-4-yl]methyl-3-pyridinecarboxamide (JNJ47965567). Therefore, the concentration-dependent increasing BzATP-evoked astroglial D-serine release was primarily generated by the activation of P2X7R. The astrocytes in S286L-TG probably also have no functional abnormalities associated with P2X7R, since the concentration-dependent responses of astroglial D-serine releases to BzATP-evoked stimulation in the wild type and S286L-TG were almost equal (Figure 7). The selective pannexin1 inhibitor, 100 μM 10PANX, also inhibited BzATP-evoked astroglial D-serine release, whereas the selective connexin43 inhibitor, 20 μM Gap19, did not affect it. These results suggest that BzATP-evoked astroglial D-serine release is mainly generated by the complex between the P2X7R and pannexin1-hemichannels.

## 3. Discussion

**Figure 8:**
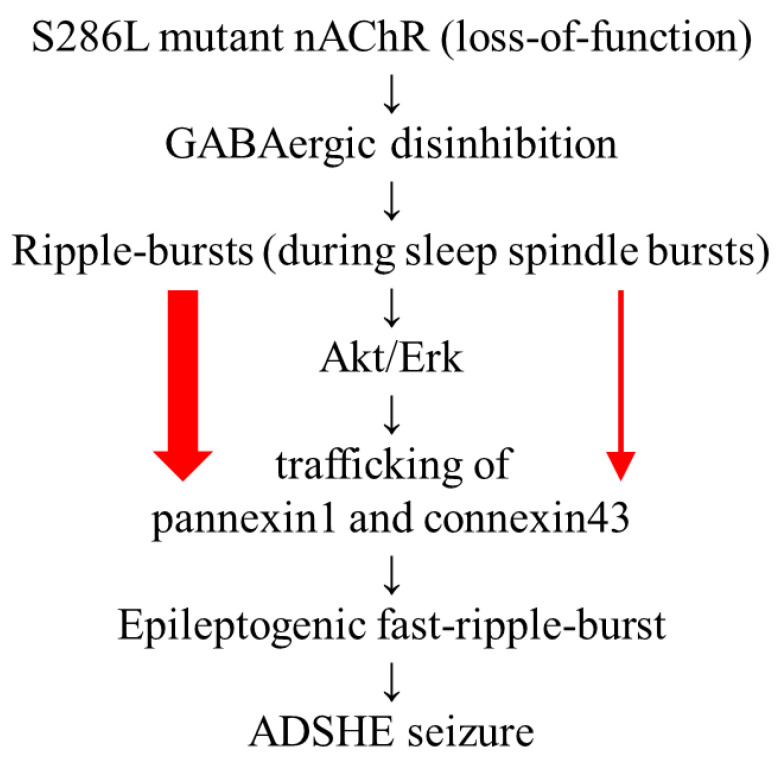
Hypothetical age-dependent pathomechanisms of ADSHE in S286L-TG. Impaired S286L-mutant nAChR leads to GABAergic disinhibition, resulting in enhanced propagation of physiological ripple burst excitabilities [8,9,10,13,14]. The hyperexcitability increases intracellular signaling of Akt and Erk, which enhance trafficking of pannexin1 and connexin43 to the astroglial plasma membrane [8,11,12]. Increased expression of pannexin1 and connexin43 is also functionally activated by ripple burst [8,11]. The activation of pannexin1-hemichannels is probably more sensitive and earlier than that of connexin43-hemichannels due to the pannexin1-hemichannel functional features, such as depolarization dependency and extracellular Ca^2+^ independency. These signaling cascades contribute to development of epileptogenic fast ripple bursts.

The present study demonstrated that any functional abnormalities in primary cultured astrocytes of S286L-TG could not be detected when compared to those of the wild type; however, the pannexin1 expression in the plasma membrane in the OFC (a major ADSHE focus region) of S286L-TG increased age-dependently. These findings suggest that enhanced astroglial transmission in the OFC of S286L-TG is not directly induced by genic abnormalities in S286L-mutant *Chrna4*; rather, they indicate that some abnormalities in tripartite synaptic transmission play important roles in the development of epileptogenesis/ictogenesis in the ADSHE of S286L-TG [8]. According to previous findings regarding the functional abnormalities in connexin43-hemichannels [9,10,11,12], the present study explored the impacts of electrophysiological excitabilities, such as physiological ripple burst and epileptogenic fast ripple bursts, on pannexin1 expression in the astroglial plasma membrane and D-serine release from cultured astrocytes. The results, which showed that pannexin1 expression in the plasma membrane in the OFC of S286L-TG age-dependently increased within a range of 4, 7, and 10 weeks of age, are also interpreted to be consistent with the age-dependent development process of epileptogenesis/ictogenesis in S286L-TG, since the onset of interictal and ictal discharges in S286L-TG was observed from 6 to 8 weeks of age and after 8 weeks of age, respectively [8,11,14]. Furthermore, the fast ripple burst occurrence was synchronized with the initiation of the ictal discharges [11]. In healthy individuals, ripple burst is considered to play an important role in the generation of several components of cognition within the thalamo-hippocampal and thalamocortical pathways [32,34,59,60]. Therefore, ripple burst, which contributes to generation of procognitive components in healthy individuals, may lead to the development of epileptogenesis/ictogenesis in individuals with ADSHE vulnerability, such as the S284L-mutant *Chrna4*, under the intrathalamic GABAergic disinhibition induced by the loss-of-function mutant nAChR [8,9,10,13,14]. These demonstrations can provide a reasonable hypothesis stating that the ripple burst during sleep spindles contributes to the development of epileptogenic fast ripple burst in S286L-TG. Additionally, a reasonable explanation for the pathomechanism by which ADSHE seizures typically occur during sleep can also be provided [61,62,63,64]. Our proposed hypothesis regarding the pathomechanisms of ADSHE in S286L-TG is described in Figure 8.

Pannexin family proteins are ubiquitously expressed in the somatic and central nervous systems, including those of neurons and astrocytes in the cortex [65,66,67]. Clinical studies revealed that the expression of pannexin1 in the focus regions of patients with temporal lobe epilepsy and focal cortical dysplasia increased [68,69]. Preclinical studies also demonstrated that cobalt-induced seizure increased pannexin1 expression, which was inhibited by tetrodotoxin via the suppression of hyper-depolarization [70]. Taken together with these previous findings, although pannexin1 is expressed ubiquitously, the present results, which demonstrate that the increasing expression of pannexin1 was observed selectively in the focus region and was temporally synchronized with the onset of interictal discharge in S286L-TG, support the derivation of pannexin1 activation by the propagation of HFOs. Both the pannexin1-hemichannels and the connexin43-hemichannels are activated by plasma membrane depolarizations, but the pannexin1-hemichannels are more sensitive to depolarization compared to the connexin43-hemichannels [71,72,73,74]. Indeed, in this study, ripple burst-evoked stimulation predominantly activated the pannexin1-hemichannels compared to the connexin43-hemichannels, whereas fast ripple burst-evoked stimulation predominantly activated the connexin43-hemichannels. These findings strongly indicated the possibility that pannexin1 might receive functional abnormalities (hyperexcitability) earlier than connexin43 at the initiation of ADSHE epileptogenesis development in S286L-TG. We shall report on this in further studies to clarify the detailed contribution of increased pannexin1-hemichannels to epileptogenesis/ictogenesis in vivo.

It has been reported that sustained/repetitive exposure to P2X7R agonists changes the features of P2X7R from cation channels to non-selective channels that are permeable to large molecules and also facilitates the complex formation of P2X7R/pannexin1 complex, which can also permeate large molecules [29,30,31]. In this study, the acute application of BzATP increased astroglial D-serine release, which was inhibited by the P2X7R antagonist, JNJ47965567, and the pannexin1-hemichannel inhibitor, 10PANX, but was not affected by the connexin43-hemichannel inhibitor, Gap19. Increased ATP release contributes to epileptic excitability; however, extracellular ATP is immediately degraded to inhibitory adenosine by ectonucleotides [75]. Adenosine metabolized from released ATP in the extracellular space activates both anticonvulsive A1 and proconvulsive A2A receptors [76,77,78]. Therefore, purinergic transmission functions are a double-edged sword in terms of the epileptogenesis/ictogenesis regulation of the excitatory/inhibitory balance of tripartite synaptic transmission [6,7,79,80]. To explore the functional abnormalities of purinergic transmission in S286L-TG, age-dependent extracellular levels of ATP and adenosine in the ADSHE focus region of S286L-TG should be determined using microdialysis. This study elucidated the fact that the activated pannexine1-hemichannel played important roles in the endogenous glutamate receptor agonistic gliotransmitter, D-serine [81,82]. The findings in this study regarding increasing astroglial D-serine release through pannexin1-hemichannels can provide some of the mechanisms of the epileptogenesis/ictogenesis of S286L-TG. To identify the more detailed pathomechanisms underlying the epileptogenesis/ictogenesis of S286L-TG, we shall report on the impacts of enhanced pannexin1-hemichannels in S286L-TG in further studies.

## 4. Materials and Methods

### 4.1. Experimental Animals

All the experimental procedures, including those for animal care and the protocols for animal experiments, were approved by the Animal Research Ethics Committee of the Mie University School of Medicine (No. 24-37-R3, 7 March 2018) and performed in accordance with the ethical guidelines established by the Institutional Animal Care and Use Committee at Mie University, Japan, and the Animal Research: Reporting of In Vivo Experiments guidelines [83].

A total of 102 male rats, wild-type littermates (n = 72), and S286L-TG (n = 30) (Sprague Dawley strain background, SLC, Shizuoka, Japan), and pregnant female wild type (n = 6) and S286L-TG (n = 3) (Sprague-Dawley rat background: SLC) were housed individually in cages and kept in air-conditioned rooms (temperature, 22 ± 2 °C) with a 12 h light/dark cycle, with ad libitum access to food and water. Neonatal rats (0–48h of age: total n = 66), including wild-type Sprague-Dawley rats (n = 54) and S286L-TG rats (n = 12), were used for culturing astrocytes in vivo.

### 4.2. Chemical Agents

The 2′(3′)-O-(4-benzoylbenzoyl)adenosine-5′-triphosphate tri(triethylammonium) salt (BzATP: potent rat P2X7R agonist) [84,85], 2-(phenylthio)-*N-*[[tetrahydro-4-(4-phenyl-1-piperazinyl)-2H-pyran-4-yl]methyl-3-pyridinecarboxamide (JNJ47965567: selective P2X7R antagonist) [86], TAT-Gap19 (Gap19: selective connexin43 inhibitor) [87], 10PANX (selective pannexin1 inhibitor) [31], probenecid (pannexin1 inhibitor) [31,47], and carbenoxolone (CBX: non-selective astroglial hemichannel inhibitor) [2] were obtained from Funakoshi (Tokyo, Japan).

All the compounds were prepared on the day of the experiment. JNJ47965567 and probenecid were initially dissolved in 1 N HCl (50 mM) and DMSO (50 mM), respectively. BzATP, Gap19, 10PANX, and CBX were dissolved directly in the experimental medium.

### 4.3. Primary Cultured Astrocytes

Cortical primary cultured astrocytes from the wild type and S286L-TG were prepared according to previous studies [11,88]. The cerebral hemispheres were then removed using a dissection microscope. The brain was chopped into fine pieces using scissors and triturated using a micropipette. The suspension was filtered through a 70 μM nylon mesh (BD, Franklin Lakes, NJ, USA) and then centrifuged. The pellets were resuspended in Dulbecco’s modified Eagle’s medium (D6546; Sigma-Aldrich, St. Louis, MO, USA) containing 10% fetal calf serum (fDMEM). After 14 days of culture (DIV14) to DIV28, the astrocytes were trypsinized and seeded directly on a translucent polyethylene terephthalate membrane (1.0 μm) in 24-well plates (BD, Franklin Lakes, NJ, USA) at density of 100 cells/cm for the experiments. fDMEM was changed twice a week between DIV14 and DIV28. On DIV28, the astrocytes were washed out using artificial cerebrospinal fluid (ACSF) (150 mM Na^+^, 3.0 mM K^+^, 1.4 mM Ca^2+^ and 0.8 mM Mg^2+^ and 5.5 mM glucose adjusted to pH = 7.3 using 20 mM HEPES buffer) for the experiments (ACSF was prepared before the experiment) [12].

### 4.4. Artificial HFO-Evoked Stimulation and BzATP-Evoked Stimulation

The accumulation of HFOs, including either physiological ripple burst or epileptogenic fast ripple burst [11,89], has been reported to play an important role in the epileptogenesis of S286L-TG [8,12,14]. HFOs have physiological and pathological/epileptogenic oscillatory activities within a limited frequency band ranging from 80 to 500 Hz that clearly stands out from the baseline and persists for at least four oscillation cycles [11,89]. HFOs are composed of two frequency ranges: a relatively slow physiological procognitive ripple burst (80–250 Hz for tens of milliseconds in duration) and an epileptogenic fast ripple burst (250–500 Hz and millisecond duration). Cultured astrocytes were activated by artificial ripple burst or fast ripple burst stimulations using a bus drive amplifier (SEG-3104 MG; Miyuki Giken, Tokyo, Japan). The ripple burst- and fast ripple burst-evoked stimulations were set at a square-wave direct-current pulse output with a magnitude of 300 mV/mm [11]. The ripple burst-evoked stimulation was composed of 10 stimuli at 200 Hz and 10 bursts (50% duty cycle) at burst intervals of 100 msec/sec [11]. A set of fast ripple-evoked stimulations was composed of 10 stimuli at 500 Hz and 10 bursts (50% duty cycle) at burst intervals of 40 msec/sec. These HFO-evoked stimulation patterns were regulated using LabChart v8.2 software (AD Instruments, Dunedin, New Zealand). The amounts of energization during the ripple burst- and fast ripple burst-evoked stimulations were set to be equal.

To explore the acute effects of HFO-evoked stimulation on astroglial D-serine release, on DIV28 after the washout, the cultured astrocytes received a set of 100 ripple-evoked or fast ripple-evoked stimulations incubated in ACSF or FC-ACSF containing target agents (20 μM Gap19, 100 μM 10PANX, 1–100 μM carbenoxolone, or 100–1000 μM probenecid). The composition of NaCl in FC-ACSF was modified to maintain isotonicity and ionic strength. To explore the chronic effects of HFO-evoked stimulation on pannexin1 expression in the astroglial plasma membrane fraction, the cultured astrocytes were received a set of 100 ripple-evoked or fast ripple-evoked stimulations for 7 days in fDMEM (DIV21–28).

To explore the acute concentration-dependent effects of BzATP on DIV28 after the washout, the cultured astrocytes were incubated in ACSF containing BzATP (1–100 μM) with or without 3 μM JNJ47965567, 20 μM Gap19, or 100 μM 10PANX.

### 4.5. Determination of Levels of D-Serine

The D-serine levels were determined using ultra-high-performance liquid chromatography (UHPLC) (PU-4185, Jasco, Tokyo, Japan) with fluorescence resonance energy transfer detection (FP-4020, Jasco), after dual derivatization with isobutyryl-L-cysteine and o-phthalaldehyde [14]. Derivative reagent solutions were prepared by dissolving isobutyryl-L-cysteine (2 mg) and o-phthalaldehyde (2 mg) in 0.1 mL of ethanol, followed by the addition of 0.9 mL of sodium borate buffer (0.2 M, pH 9.0) [14]. Automated pre-column derivatives were prepared by drawing up a 5 μL aliquot of the sample, standard, or blank solution and 5 μL of the derivative reagent solution and allowing the two to react in reaction vials for 5 min before injection. The derivatized samples (5 μL) were injected using an autosampler (AS-4150, Jasco). The analytical column (Triat C18, particle 1.8 μM, 50 × 2.1 mm, YMC, Kyoto, Japan) was maintained at 45 °C, with the flow rate set at 500 μL/min. A linear gradient elution program was performed over 10 min with mobile phases A (0.05 M citrate buffer, pH 5.0) and B (0.05 M citrate buffer containing 30% acetonitrile and 30% methanol, pH 3.5). The excitation/emission wavelengths of the fluorescence detector were set at 345/455 nm [14]. To correct the deviations of the D-serine level due to the cultured cell number, the total protein level was determined after the experiments using a protein assay reagent kit (FUJIFILM Wako Pure Chemical Corporation; Osaka, Japan) [14].

### 4.6. Capillary Immunoblotting

The orbitofrontal cortex (OFC) of S286L-TG and the wild type was removed, and their plasma membrane fractions were extracted using the Minute Plasma Membrane Protein Isolation Kit (Invent Biotechnologies, Plymouth, MN, USA). On DIV28, after the washout, the plasma membrane fraction of the cultured astrocytes was also extracted using the Minute Plasma Membrane Protein Isolation Kit.

A capillary immunoblotting analysis was performed using Wes (ProteinSimple, Santa Clara, CA, USA) [90] according to the ProteinSimple user manual. The plasma membrane fractions were mixed with a master mix (ProteinSimple) until the final concentration of 1 × sample buffer, 1 × fluorescent molecular weight marker, and 40 mM of dithiothreitol was obtained; then, they were heated at 95 °C for 5 min. The samples, blocking reagent, primary antibodies, horseradish peroxidase (HRP) conjugated secondary antibody, chemiluminescent substrate (SuperSignal West Femto; Thermo Fisher Scientific, Waltham, MA, USA), and separation and stacking matrices were also distributed into designated wells in a 25-well plate. After plate loading, separation electrophoresis and immunodetection were performed in a capillary system, which was fully automated. Capillary immunoblotting was performed at room temperature using the default settings of the instrument. The capillaries were first filled with a separation matrix, followed by a stacking matrix and a sample loading of approximately 40 nL. During electrophoresis, the proteins were separated based on molecular weight through stacking and separation matrices at 250 V for 40 min and then immobilized on the capillary wall using proprietary photoactivated capture chemistry. The matrices were then washed again. Next, the capillaries were incubated with a blocking reagent for 15 min and the target proteins were probed with primary antibodies, followed by incubation with HRP-conjugated secondary antibodies (anti-rabbit HRP-conjugated IgG, A00098, 10 μg/mL, GenScript, Piscataway, NJ, USA). The antibodies against GAPDH (NB300–322, 1:100, Novus Biologicals, Littleton, CO, USA) and pannexin1 (12595-1-AP, 1:100, Proteintech, Rosemont, IL, USA) were diluted in Immuno Shot Platinum (CosmoBio, Tokyo, Japan).

### 4.7. Data Analysis

All the experiments were designed with groups containing equal numbers of animals (n = 6), without a formal power analysis, in accordance with previous studies. All the values are expressed as the mean ± standard deviation (SD), and *p* values of <0.05 (two-tailed) were considered statistically significant in all the tests.

The levels of drugs for administration were selected based on the values reported in previous studies. Where possible, we aimed to randomize and blind the data. To determine the levels of D-serine and pannexin1, the sample order of the autosamplers was selected using random number tables.

The age-dependent fluctuation of pannexin1 expression between the wild type and S286L-TG was analyzed using two-way analysis variance (ANOVA) with Scheffe’s post hoc test using BellCurve for Excel version 3.2 (Social Survey Research Information Co., Ltd., Tokyo, Japan). The chronic effects of HFO-evoked stimulation on pannexin1 expression in the wild type and S286L-TG were also analyzed using two-way analysis variance (ANOVA) with Scheffe’s post hoc test. The use-dependent effects of HFO-evoked stimulations on D-serine release from the cultured astrocytes in the wild type and S286L-TG were also analyzed using two-way ANOVA with Scheffe’s post hoc test. The effects of the extracellular Ca^2+^ level and the inhibitors of pannexin1 and connexin43 on HFO-evoked D-serine release from the cultured astrocytes of the wild type were analyzed using multivariate analysis of variance (MANOVA) with Scheffe’s post hoc test using BellCurve for Excel version 3.2. When the data did not violate the assumption of sphericity (*p* > 0.05), the F value of the MANOVA was analyzed using assumed degrees of freedom of sphericity. When the assumption of sphericity was violated (*p* < 0.05), the F value was analyzed using Chi-Muller-corrected degrees of freedom. When the F value of the MANOVA was significant, the data were analyzed using Scheffe’s post hoc test.

## 5. Conclusions

The present study demonstrated several candidate pathomechanisms of ADSHE associated with pannexin1-hemichannels using S286L-TG. It has been already revealed that before the onset of ADSHE seizure in S286L-TG (during 4–8 weeks of age), the physiological ripple burst contributes to the development of epileptogenic fast ripple burst induced by the enhancement of the trafficking of connexin43 to the plasma membrane via the activation of the intracellular signaling of Akt and Erk [8,9,10,11,12,13,14]. In an in vivo study using S286L-TG, no abnormalities of pannexin1 expression in the ADSHE focus region (OFC) of S286L-TG at 4 weeks of age (before the onset of epileptic discharge) were observed; however, at 7 (critical period of onset of interictal discharge) and 10 (after the onset of ADSHE seizures) weeks of age, the pannexin1 expression age-dependently increased. In in vitro experiments using cultured astrocytes of both the wild type and S286L-TG, the transmission function of the astrocytes from S286L-TG did not differ from that of the astrocytes from the wild type; this was similar to that of the connexin43-hemichannels [8,11,12]. However, the application of chronic HFO-evoked stimulation increased pannexin1 expression in the plasma membrane. These demonstrations regarding the age-dependent activation of the astroglial pannexin1-hemichannel function in S286L-TG were observed to be similar to the temporal patterns in the astroglial connexin43-hemichannels. Therefore, these results suggest that the hyperactivation of both ripple burst and fast ripple burst under the GABAergic disinhibition is, at least partially, involved in the development of the epileptogenesis/ictogenesis of ADSHE in S286L-TG through increasing pannexin1 expression in the astroglial plasma membrane.

## Data Availability

The data that support the findings of this study are available from the corresponding author upon reasonable request. Some data may not be made available due to ethical restrictions.
